# Use of liposomal irinotecan with 5-FU and oxaliplatin (NALIRIFOX) in neoadjuvant pancreatic adenocarcinoma: NEO-Nal-IRI trial

**DOI:** 10.1093/oncolo/oyag250

**Published:** 2026-06-29

**Authors:** Thomas J George, Tuo Lin, Ibrahim Nassour, Sherise C Rogers, Tim Jang, Ilyas Sahin, Brian H Ramnaraign, Jesus Fabregas, Kathryn Hitchcock, Gonghao Liu, Karen B Russell, Anita A Turk, Omar Kayaleh, Z Hugh Fan, David L DeRemer, Ryan M Thomas, Ji-Hyun Lee, Steven J Hughes

**Affiliations:** Department of Medicine, University of Florida, Gainesville, FL 32610, United States; Department of Biostatistics and UF Health Cancer Institute, Gainesville, FL 32610, United States; Department of Surgery, University of Florida, Gainesville, FL 32610, United States; Department of Medicine, University of Florida, Gainesville, FL 32610, United States; Department of Medicine, University of Florida, Gainesville, FL 32610, United States; Department of Medicine, University of Florida, Gainesville, FL 32610, United States; Department of Medicine, University of Florida, Gainesville, FL 32610, United States; Department of Medicine, University of Florida, Gainesville, FL 32610, United States; Department of Radiation Oncology, University of Florida, Gainesville, FL 32610, United States; Department of Biostatistics and UF Health Cancer Institute, Gainesville, FL 32610, United States; Tallahassee Memorial Hospital, Tallahassee, FL 32308, United States; Department of Medicine, Indiana University Simon Comprehensive Cancer Center, Indianapolis, IN 46202, United States; Orlando Health Cancer Institute, Orlando, FL 32806, United States; Department of Mechanical and Aerospace Engineering, College of Engineering, University of Florida, Gainesville, FL 32610, United States; Department of Pharmacotherapy and Translational Research, College of Pharmacy, University of Florida, Gainesville, FL 32610, United States; Department of Surgery, University of Florida, Gainesville, FL 32610, United States; Department of Biostatistics and UF Health Cancer Institute, Gainesville, FL 32610, United States; Department of Surgery, University of Florida, Gainesville, FL 32610, United States

**Keywords:** pancreatic ductal adenocarcinoma, liposomal irinotecan, NALIRIFOX, resectable and borderline resectable pancreatic cancer, phase II clinical trial, total neoadjuvant therapy

## Abstract

**Background:**

While neoadjuvant FOLFIRINOX is an effective regimen for pancreatic ductal adenocarcinoma (PDAC), toxicity frequently limits its use. Nanoliposomal irinotecan (nal-IRI) offers improved pharmacokinetic properties and may mitigate some of the side effects. We conducted a multi-institutional, phase II study to evaluate the safety and clinical activity of neoadjuvant NALIRIFOX (nal-IRI, 5-fluorouracil, leucovorin, and oxaliplatin) in patients with resectable and borderline resectable (R/BR) PDAC.

**Methods:**

Patients with untreated R/BR PDAC received 8 cycles of neoadjuvant NALIRIFOX. The primary endpoint was the 30-day post-operative major complication rate among resected patients. Secondary endpoints included treatment completion rate, R0 resection rate, objective response rate (ORR), biochemical (CA19-9) and radiographic responses, nodal downstaging, and quality of life (QoL by FACT-G).

**Results:**

Of the 45 enrolled patients, 73% completed all 8 cycles of neoadjuvant NALIRIFOX. 34 patients (76%) underwent attempted surgery, of whom 29 (64%) had complete resection (15 BR patients and 14 R patients). The 30-day post-operative major complication rate was 10% (95% CI, 2.2-27%, *P* = .012), meeting the pre-specified threshold. R0 resection was achieved in 90% of resected patients. The radiographic ORR was 45% (95% CI, 29-62%), and the clinical benefit rate was 73% (95% CI, 58-85%).

**Conclusion:**

Neoadjuvant NALIRIFOX is a safe and active regimen in R/BR PDAC with a low post-operative complication rate, high treatment completion and R0 resection rates, and meaningful clinical responses. These findings support further investigation of NALIRIFOX as part of a total neoadjuvant therapy approach in PDAC. [ClinicalTrials.gov identifier: NCT03483038].

Lessons Learned

- Neoadjuvant NALIRIFOX is safe and efficacious in R/BR PDAC, meeting the primary endpoint of a low 30-day major complication rate (10%) and showing promising results across multiple secondary endpoints as above.
- Neoadjuvant NALIRIFOX has a low perioperative risk with a tolerable side profile while able to provide meaningful disease control and preserve surgical outcomes.
- The pharmacokinetic and toxicity profile of nanoliposomal irinotecan permits effective delivery of preoperative systemic therapy; This supports further investigation of NALIRIFOX, potentially in a total neoadjuvant therapy framework for R/BR PDAC.

## Trial information

**Table:** 

**Trial information**	
**Disease**	Pancreatic ductal adenocarcinoma (PDAC)
**Stage of disease/treatment**	Resectable and borderline resectable PDAC without metastasis
**Prior therapy**	None
**Type of study**	Phase II
**Primary endpoints**	30-day postoperative major complication rate among resected patients
**Secondary endpoints**	Toxicity, treatment completion rate, R0 resection rate, clinical benefit rate, radiographic response, CA19-9 response; quality of life assessment using FACT-G validated patient-reported outcome instrument.
**Additional details of endpoints or study design** **Participants received 8 cycles (4 months) of neoadjuvant NALIRIFOX if tolerated followed by restaging and surgical evaluation. Preoperative radiation and postoperative therapy were permitted at investigator discretion. All subjects who were given at least one dose of the neoadjuvant NALIRIFOX were included in the analysis for a modified intention-to-treat (mITT) approach.**	

## Drug information

**Table:** 

**Drug information**	
**Generic/working name**	Nanoliposomal irinotecan (nal-IRI)
**Company name**	Ipsen
**Drug type**	Chemotherapy
**Drug class**	Topoisomerase 1 inhibitor
**Dose**	50 mg/m²
**Route**	IV
**Schedule of administration**	Day 1 of 14-day cycle
**Drug information**	
**Generic/working name**	Oxaliplatin
**Company name**	Multiple manufacturers
**Drug type**	Chemotherapy
**Drug class**	Alkylating agent
**Dose**	60 mg/m²
**Route**	IV
**Schedule of administration**	Day 1 of 14-day cycle
**Drug information**	
**Generic/working name**	5-Fluorouracil (5-FU)
**Company name**	Multiple manufacturers
**Drug type**	Chemotherapy
**Drug class**	Antimetabolite
**Dose**	2400 mg/m²
**Route**	IV (Continuous infusion)
**Schedule of administration**	Continuous infusion over 46 hours starting Day 1 of a 14-day cycle

**Table:** 

**Drug information**	
**Generic/working name**	Leucovorin (Folinic acid)
**Company name**	Multiple manufacturers
**Drug type**	Vitamin
**Drug class**	Folinic acid analog
**Dose**	400 mg/m²
**Route**	IV
**Schedule of administration**	Day 1 of a 14-day cycle

**Table:** 

**Patient characteristics**	
**Number of patients, male**	21
**Number of patients, female**	24
**Stage**	I-III
**Resectability:**	
** Resectable**	15
** Borderline resectable**	30
**Number of prior systemic therapies: median (range)**	0
**Performance status: ECOG 0 or 1**	45
**Performance status: ECOG 2 or above**	0
**Cancer types or histologic subtypes**	Pancreatic ductal adenocarcinoma

Between May 2019 and February 2023, 45 patients were enrolled from 4 centers in the U.S.. The median age was 63 (range 41 to 76). The majority of the enrolled patients were White (89%) and women (53%) and had borderline (67%) or resectable (33%) disease at study entry confirmed by a multidisciplinary consensus conference. Initial biopsy tissues from 24 patients had adequate samples for molecular profiling. Of those, KRAS mutations were identified in 23 patients and TP53 mutations in 17 patients, with both alterations distributed in similar proportions between BR and R patients. NTRK mutation was identified in one patient with BR disease. RET mutation and microsatellite instability were each identified in one patient with R disease. Otherwise, the testing was negative for other actionable mutations including HER2 and BRAF.

## Primary assessment method

**Table:** 

**Primary/secondary assessment method**	
**Endpoints**	Primary endpoint: 30-day post-operative major complication rate among patients who underwent pancreatic tumor resection. Secondary endpoint: Toxicity, treatment completion, rates of R0 resection, clinical, biochemical, and radiographic response, QOL.
**Number of patients screened**	45
**Number of patients enrolled**	45
**Number of patients evaluable for toxicity**	45
**Number of patients evaluated for efficacy**	29
**Evaluation method**	Using a modified intention to treat (mITT) analysis, approximately 28 patients were estimated to be required (accounting for an anticipated 10% dropout rate) to achieve 80% power to detect a 20% reduction in the 30-day complication rate, from 30% to 10%, using a one-sided exact test with a significance level of 0.05with 95% confidence intervals (CIs) calculated using the binomial distribution. Statistical significance was defined as a *P*-value < .05. Statistical analyses were conducted using R v4.1.
**Outcome notes** **The majority of patients who started therapy (*n* = 34; 76%) proceeded to surgery, and 29 patients (15 borderline resectable and 14 resectable) completed definitive resection and were thus evaluable (Figures 1 and 2). The study met the primary endpoint with a higher than anticipated treatment completion rate and favorable surgical outcomes for this relatively high-risk cohort. Thirty-day post-operative major complications were observed in 10% of patients (95% CI, 2.2-27%, *P* = .012; Figure S2).** **NALIRIFOX was well tolerated by enrolled patients, with 73% (33/45) of enrolled patients completing all 8 cycles of planned neoadjuvant NALIRIFOX. The reported adverse events were mostly grade 1 or 2 in severity without new safety signals. There were no treatment-related deaths from the neoadjuvant therapy. The QoL assessment through FACT-G showed stable scores across all domains with minimal fluctuations between each cycle (Figure S1).** **The radiographic ORR following neoadjuvant NALIRIFOX was 45% (95% CI: 29-62%) with 73% (95% CI: 58-85%) achieving a clinical benefit (CR + PR + SD) (Figure S3). A log transformation was utilized for clarity due to high variability in the data (Figure 3), and it showed a CA 19-9 reduction in most patients.**	

The majority of patients who started therapy (n=34; 76%) proceeded to surgery, and 29 patients (15 borderline resectable and 14 resectable) completed definitive resection and were evaluable. The study met the primary endpoint with a higher than anticipated treatment completion rate and favorable surgical outcomes for this relatively high-risk cohort. Thirty-day post-operative major complications were observed in 10% of patients (95% CI, 2.2 to 27%, p=0.012) (Supplemental Figure S2). 30-day postoperative major complication rates were similar regardless of resectability status at diagnosis, surgical procedure type, extent of nodal involvement on final pathology, or histologic tumor grade. Moreover, dose reductions, treatment delays, or agent discontinuation did not correlate with higher 30-day postoperative major complication rates among resected patients in this study. These major complications included GI bleeding, ruptured pseudoaneurysm, and acute hemorrhagic shock. The radiographic ORR following neoadjuvant NALIRIFOX was 45% (95% CI: 29–62%) with 73% (95% CI: 58–85%) achieving a clinical benefit (CR + PR + SD) (Supplemental Figure S3). For those completing surgery, the R0 resection rate was 90% with 7% and 3% of patients achieving R1 and R2 resections, respectively (Figure 2). Higher R0 resections were observed among patients with resectable disease at presentation, ≤1 positive lymph node, and well- or moderately differentiated tumors, although these differences did not reach statistical significance. Moreover, R0 resection was achieved at comparably high rates independent of KRAS or TP53 mutation status, with consistently low and similar frequencies of margin-positive resections observed between KRAS-mutant and KRAS-wild-type tumors, as well as between TP53-mutant and TP53-wild-type tumors. Nodal downstaging was noted in a large proportion of patients following neoadjuvant NALIRIFOX. Matched radiographic assessments in resected patients revealed that 26 out of 29 patients (90%) exhibited nodal disease control (stable or improved nodal status) following treatment with NALIRIFOX. Pathological evaluation revealed stable or improved nodal status in 14 of 29 patients (48%) when compared to clinical staging at diagnosis. Tumor regression grade (TRG) was available for 32 out of 34 patients (94%) following resection. At the time of analysis, 11 patients (34%) were alive, 20 (63%) were deceased, and 1 patient (3%) had an unknown survival status. Survival outcomes varied by TRG category— Patients with TRG 1 demonstrated the most favorable outcomes, with 2 of 3 patients (67%) alive at last follow-up. Survival decreased with worsening TRG: 8 of 19 patients (42%) with TRG 2 were alive, compared with only 1 of 10 patients (10%) with TRG 3, suggesting worse survival outcomes with higher TRG. There were no pathologic complete responses reported.

The median CA19-9 at baseline for the entire cohort was 110 (range 0-20,000). The median CA19-9 reduction across the cohort was 29.0 (range: 0–19,434), with a mean of 756.9 (SD: 2,932.7). A log transformation was utilized for clarity due to high variability in the data (Figure 3), and it showed a CA 19-9 reduction in most patients. No clear correlation was observed between the reduction in CA 19-9 at multiple time points and downstaging or R0 resection rates. An exploratory biomarker analysis revealed that among the 38 patients with a decrease in CA 19-9 from baseline, 23 (60%) proceeded to surgery, with 20 achieving R0 resection, 2 R1 resection, and 1 R2 resection. In contrast, 6 of the 7 patients without a decrease in CA 19-9 proceeded to surgery and all achieved a R0 resection.

**Figure 1.**
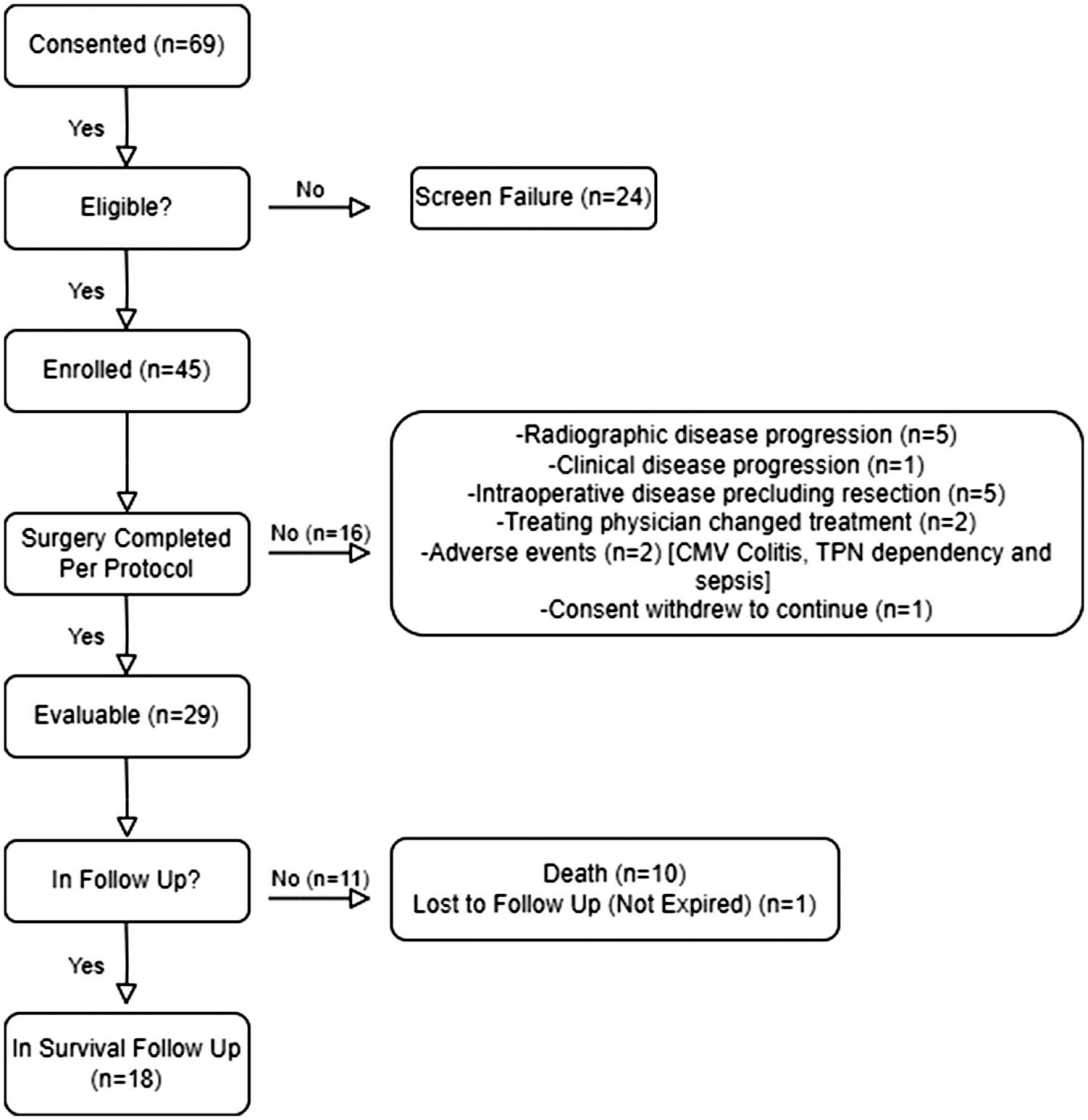
CONSORT flow diagram for all patients enrolled. CMV: Cytomegalovirus; TPN: Total Parenteral Nutrition.

**Figure 2.**
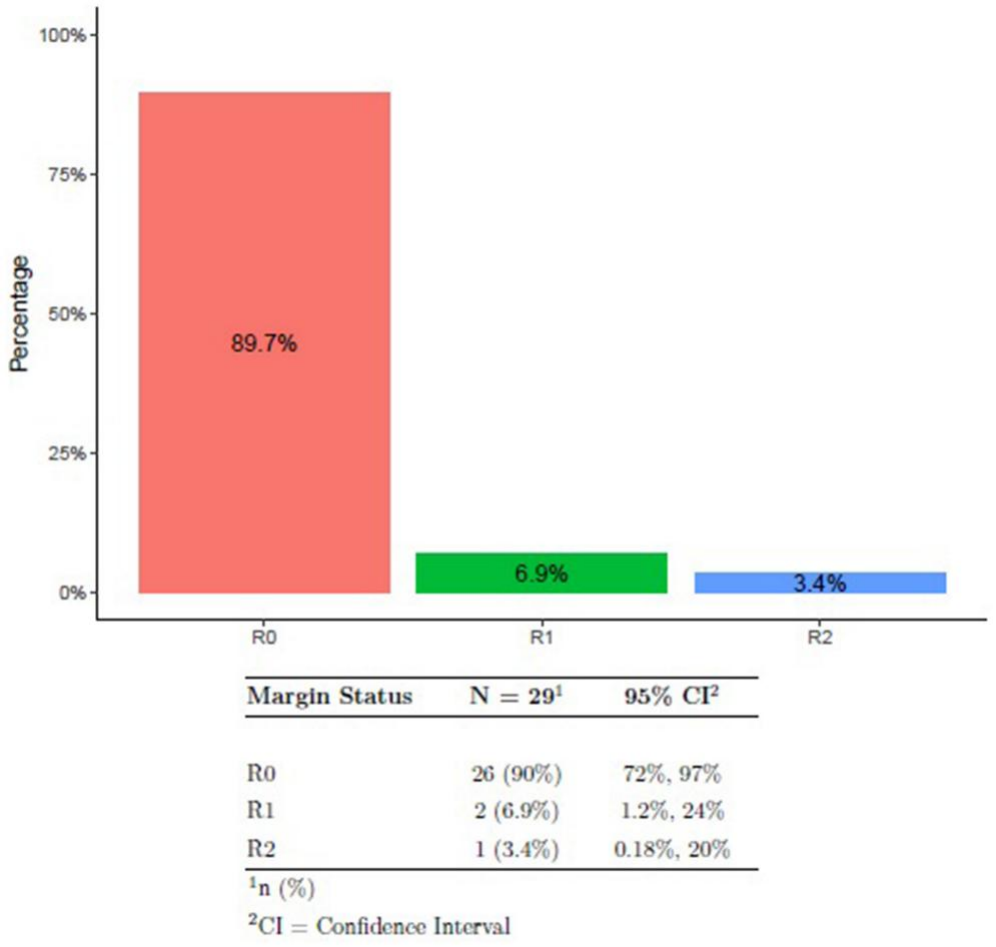
The surgical complete resection rate (R0) according to the review of the final pathology reports, pathological nodal status and TNM staging (AJCC 8th Edition). Calculation of CI was done using Chi-square test.

**Figure 3.**
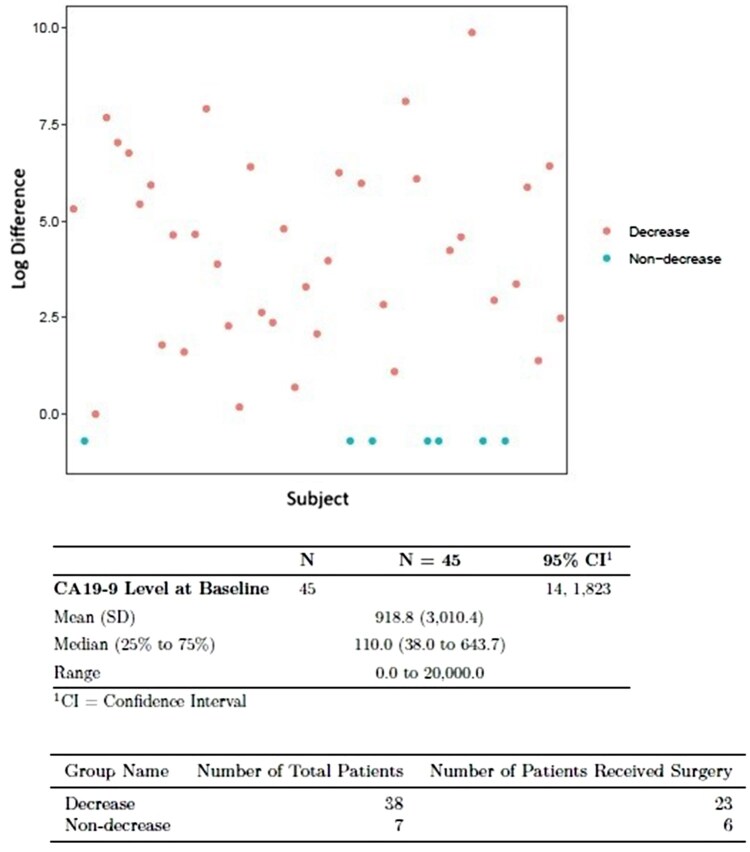
Biochemical response/recurrence rate is measured as the difference between baseline and lowest point of serum CA19-9 level prior to surgery. A log transformation applied to minimize the high variability of the data.

**Table:** 

Table 2. Serious adverse events		
Toxicity category	CTCAE toxicity term	Overall count (%)
**Blood and lymphatic system disorders**	Febrile neutropenia	2 (5.41%)
	Anemia	2 (5.41%)
**Gastrointestinal disorders**	Diarrhea	11 (29.73%)
	Nausea	8 (21.62%)
	Vomiting	6 (16.22%)
	Abdominal pain	3 (8.11%)
	Pancreatitis	2 (5.41%)
	Gastroparesis	1 (2.7%)
	Mucositis oral	1 (2.7%)
	Colitis	1 (2.7%)
	Lower gastrointestinal hemorrhage	1 (2.7%)
**General disorders and administration site conditions**	Fatigue	7 (18.92%)
	General disorders, specify	1 (2.7%)
**Hepatobiliary disorders**	Hepatobiliary disorders, specify	2 (5.41%)
**Infections and infestations**	Biliary tract infection	1 (2.7%)
	Lung infection	1 (2.7%)
	Enterocolitis infectious	1 (2.7%)
	Sepsis	1 (2.7%)
	Wound infection	1 (2.7%)
	Appendicitis	1 (2.7%)
**Injury, poisoning, and procedural complications**	Postoperative hemorrhage	1 (2.7%)
	Fall	1 (2.7%)
**Investigations**	Neutrophil count decreased	15 (40.54%)
**Metabolism and nutrition disorders**	Anorexia	8 (21.62%)
	Hypokalemia	7 (18.92%)
	Hyperglycemia	4 (10.81%)
	Hyponatremia	3 (8.11%)
	Dehydration	2 (5.41%)
	Hypoglycemia	1 (2.7%)
**Musculoskeletal and connective tissue disorders**	Pain in extremity	1 (2.7%)
**Psychiatric disorders**	Confusion	1 (2.7%)
**Renal and urinary disorders**	Acute kidney injury	1 (2.7%)
**Vascular disorders**	Thromboembolic event	5 (13.51%)
	Hypertension	3 (8.11%)
	Hypotension	2 (5.41%)
	Hematoma	1 (2.7%)

Any grade 3 or higher adverse event per CTCAE is considered a serious AE.

Thirty-day post-operative major complications were observed in 10% of patients (95% CI, 2.2 to 27%, p=0.012) (Supplemental Figure S2). 30-day postoperative major complication rates were similar regardless of resectability status at diagnosis, surgical procedure type, extent of nodal involvement on final pathology, or histologic tumor grade. Moreover, dose reductions, treatment delays, or agent discontinuation did not correlate with higher 30-day postoperative major complication rates among resected patients in this study. These major complications included GI bleeding, ruptured pseudoaneurysm, and acute hemorrhagic shock.

## Table 3. General toxicity profile

**Table:** Overall, NALIRIFOX was well tolerated by enrolled patients, with 73% (33/45) of enrolled patients completing all eight cycles of planned neoadjuvant NALIRIFOX. There were no treatment-related deaths from the neoadjuvant therapy. The two patients who failed to proceed to surgery due to adverse events included CMV colitis with a resultant requirement for TPN and another patient with biliary sepsis from a failed stent. The QoL assessment through FACT-G showed stable scores across all domains with minimal fluctuations between each cycle (Supplemental Figure S1), suggesting the treatment was generally well-tolerated with minimal impact on overall QoL throughout the study. Overall, there were no new toxicities identified that were not previously reported or anticipated with this regimen.

Toxicity category	CTCAE toxicity term	Overall count
**Blood and lymphatic system disorders**	Anemia	11 (24.44%)
	Febrile neutropenia	2 (4.44%)
**Cardiac disorders**	Sinus tachycardia	3 (6.67%)
	Chest pain—cardiac	1 (2.22%)
**Eye disorders**	Blurred vision	1 (2.22%)
	Eye disorders—Other, specify	1 (2.22%)
**Gastrointestinal disorders**	Nausea	40 (88.89%)
	Diarrhea	37 (82.22%)
	Abdominal pain	22 (48.89%)
	Vomiting	19 (42.22%)
	Constipation	14 (31.11%)
	Mucositis oral	12 (26.67%)
	Dyspepsia	5 (11.11%)
	Gastrointestinal disorders—Other, specify	4 (8.89%)
	Gastroesophageal reflux disease	2 (4.44%)
	Pancreatitis	2 (4.44%)
	Stomach pain	2 (4.44%)
	Dry mouth	2 (4.44%)
	Bloating	2 (4.44%)
	Gastroparesis	1 (2.22%)
	Hemorrhoids	1 (2.22%)
	Colitis	1 (2.22%)
	Lower gastrointestinal hemorrhage	1 (2.22%)
	Abdominal distension	1 (2.22%)
	Ileus	1 (2.22%)
	Flatulence	1 (2.22%)
	Oral dysesthesia	1 (2.22%)
**General disorders and administration site conditions**	Oral pain	1 (2.22%)
	Fatigue	36 (80%)
	Edema limbs	7 (15.56%)
	Fever	6 (13.33%)
	Chills	4 (8.89%)
	Infusion related reaction	3 (6.67%)
	Facial pain	2 (4.44%)
	Non-cardiac chest pain	2 (4.44%)
	Malaise	2 (4.44%)
	Edema face	1 (2.22%)
	Flu-like symptoms 1 (2.22%)	1 (2.22%)
	General disorders and administration site conditions—Other, specify	1 (2.22%)
**Hepatobiliary disorders**	Hepatobiliary disorders—Other, specify	3 (6.67%)
	Allergic reaction	1 (2.22%)
**Immune system disorders**	Urinary tract infection	3 (6.67%)
**Infections and infestations**	Sinusitis	3 (6.67%)
	Biliary tract infection	2 (4.44%)
	Skin infection	2 (4.44%)
	Mucosal infection	1 (2.22%)
	Bronchial infection	1 (2.22%)
	Lung infection	1 (2.22%)
	Enterocolitis infectious	1 (2.22%)
	Sepsis	1 (2.22%)
	Wound infection	1 (2.22%)
	Appendicitis	1 (2.22%)
**Injury, poisoning, and procedural complications**	Bruising	2 (4.44%)
	Postoperative hemorrhage	1 (2.22%)
	Fall	1 (2.22%)
**Investigations**	Neutrophil count decreased	24 (53.33%)
	Weight loss	11 (24.44%)
	White blood cell decreased	9 (20%)
	Platelet count decreased	7 (15.56%)
	Aspartate aminotransferase increased	7 (15.56%)
	Alanine aminotransferase increased	6 (13.33%)
	Alkaline phosphatase increased	5 (11.11%)
	Lymphocyte count decreased	5 (11.11%)
	Blood bilirubin increased	3 (6.67%)
	Electrocardiogram QT corrected interval prolonged	1 (2.22%)
	Creatinine increased	1 (2.22%)
**Metabolism and nutrition disorders**	Cardiac troponin I increased	1 (2.22%)
	Hypokalemia	22 (48.89%)
	Anorexia	20 (44.44%)
	Dehydration	8 (17.78%)
	Hyperglycemia	7 (15.56%)
	Hypoalbuminemia	7 (15.56%)
	Hypomagnesemia	5 (11.11%)
	Hyponatremia	4 (8.89%)
	Hypocalcemia	3 (6.67%)
	Hypoglycemia	2 (4.44%)
	Metabolism and nutrition disorders—Other, specify	1 (2.22%)
	Hypophosphatemia	1 (2.22%)
**Musculoskeletal and connective tissue disorders**	Back pain	4 (8.89%)
	Bone pain	2 (4.44%)
	Generalized muscle weakness	2 (4.44%)
	Pain in extremity	2 (4.44%)
**Nervous system disorders**	Peripheral sensory neuropathy	24 (53.33%)
	Dysgeusia	12 (26.67%)
	Paresthesia	12 (26.67%)
	Dizziness	7 (15.56%)
	Peripheral motor neuropathy	3 (6.67%)
	Presyncope	1 (2.22%)
	Lethargy	1 (2.22%)
	Headache	1 (2.22%)
**Psychiatric disorders**	Anxiety	8 (17.78%)
	Insomnia	6 (13.33%)
	Depression	4 (8.89%)
	Agitation	2 (4.44%)
	Confusion	1 (2.22%)
**Renal and urinary disorders**	Acute kidney injury	2 (4.44%)
	Urinary tract pain	1 (2.22%)
	Hematuria	1 (2.22%)
	Urinary frequency	1 (2.22%)
**Respiratory, thoracic, and mediastinal disorders**	Dyspnea	5 (11.11%)
	Epistaxis	3 (6.67%)
	Hiccups	3 (6.67%)
	Cough	2 (4.44%)
	Nasal congestion	1 (2.22%)
	Sore throat	1 (2.22%)
	Allergic rhinitis	1 (2.22%)
**Skin and subcutaneous tissue disorders**	Alopecia	7 (15.56%)
	Pruritus 2 (4.44%)	2 (4.44%)
	Rash maculo-papular 2 (4.44%)	2 (4.44%)
	Erythroderma 1 (2.22%)	1 (2.22%)
	Skin and subcutaneous tissue disorders—Other, specify	1 (2.22%)
	Palmar-plantar erythrodysesthesia syndrome	1 (2.22%)
	Nail discoloration	1 (2.22%)
**Vascular disorders**	Thromboembolic event	7 (15.56%)
	Hypertension	5 (11.11%)
	Hypotension	3 (6.67%)
	Hematoma	2 (4.44%)
	Flushing	1 (2.22%)

If a subject had more than one count for the same toxicity, they were counted once for that toxicity

## Discussion

This phase II study demonstrated that 4 months of neoadjuvant NALIRIFOX is a feasible and safe in the preoperative treatment of potentially curable (resectable and borderline resectable) PDAC with considerable rates of radiographic, biomarker, and downstaging. These results support a growing body of evidence in the use of multi-agent chemotherapy in the neoadjuvant setting for PDAC, especially given the limited survival associated with this disease and the high rates of post-op recurrence^1–8^.

This study successfully met the primary endpoint of demonstrating a 30-day post-operative major complication rate of <10%, which is considerably lower than the anticipated rate of 30% for this group of patients with both resectable and borderline resectable disease treated across a multi-institutional collaborative of academic and community-based practices. Compared with SWOG1505, a trial that explored only 3 neoadjuvant months of either mFOLFIRINOX or gemcitabine and nab-paclitaxel for resectable PDAC,^9^^,^^10^ our study demonstrated comparable R0 resection rate, preoperative treatment completion rate, and toxicities of neoadjuvant NALIRIFOX to those of mFOLFIRINOX. Moreover, our secondary endpoint analyses compare favorably to the SWOG1505 mFOLFIRINOX treatment arm with regards to ORR (45% vs 9%) and R0 resection rate (90% vs 85%). Approximately half of patients with BR disease at diagnosis were successfully downstaged and became operable for surgical resection, demonstrating the regimen’s clinical efficacy. These findings denote that neoadjuvant NALIRIFOX can achieve meaningful downstaging of PDAC and improve surgical outcomes. Of note, while nodal status appeared stable or improved on imaging in 90% of patients after neoadjuvant NALIRIFOX, only 48% were confirmed as such on final pathology likely due to the limitations of radiographic assessment^11^^,^^12^.

Neoadjuvant NALIRIFOX demonstrated a favorable safety profile with most adverse events being mild (grades 1 or 2) and without any new or otherwise unexpected toxicities^13–16^. Manageable grade 3 and 4 adverse events were noted in this study and no grade 5 events were observed. Notably, 73% of patients completed all 8 planned cycles, supporting the feasibility of delivering 4 months of NALIRIFOX preoperatively^14^^,^^16^^,^^17^. The reported toxicities are broadly consistent with the toxicity profile reported in the NAPOLI-1 and NAPOLI-3 trials, which investigated NALIRIFOX in 1 L metastatic PDAC and the toxicity profile of perioperative NALIRIFOX was broadly comparable to pooled adverse event rates reported in a patient-level meta-analysis of neoadjuvant FOLFIRINOX in BR PDAC. Gastrointestinal toxicities were, however, more pronounced with NALIRIFOX, with grade ≥3 diarrhea and nausea occurring in 29.7% and 21.6% of patients, respectively, compared to pooled rates of 11.1% and 7.0% reported with neoadjuvant FOLFIRINOX.^18^ These rates of diarrhea and nausea were largely similar, but numerically better, in our study. In addition, FACT-G remained stable throughout treatment, reinforcing the regimen’s tolerability not only by clinical criteria but also from the patient perspective. In aggregate, our findings support continued investigation of neoadjuvant NALIRIFOX as a strategy to optimize systemic disease control while maintaining tolerability and preserving operative candidacy.

Studies demonstrating the survival advantage of adjuvant chemotherapy in pancreatic cancer included up to 6 months of FOLFIRINOX chemotherapy.^19^ In developing neoadjuvant treatment plans, the majority of contemporary trials have utilized 2, 3, or 4 months of treatment, relegating the remainder to the post-operative setting.^10^^,^^20^^,^^21^ A commonly cited rationale for limiting pre-operative systemic therapy course is to ensure patients do not miss a window of opportunity to proceed to curative intent surgery or lose functional status that jeopardizes their surgical recovery. However, adjuvant therapy is a challenge to administer and associated with increased toxicities, thus an ideal situation is to be able to administer all systemic therapy successfully in a total neoadjuvant manner, leaving surgery as a consolidative final treatment. Thus, we believe our study supports tolerability, toxicity, and outcomes data that could support a paradigm where all 6 months of systemic therapy are administered in a total neoadjuvant therapy (TNT) fashion utilizing NALIRIFOX with subsequent studies planned.

Despite the promising safety, feasibility, and surgical outcomes, there are limitations to our study. The single-arm phase II study design precludes comparison to a contemporary control group for direct outcome evaluation. This does limit our ability to attribute the observed outcomes to NALIRIFOX alone or address the impact of patient selection on the results. However, the enrollment of both resectable and borderline resectable patients with PDAC, confirmed by institutional tumor boards with centralized imaging review in this multi-institutional collaborative study across diverse clinical settings contributes to some generalizability of the outcomes and study rigor. Indeed, having both academic and community practices involved in this trial participation lends further strength to the robustness of the results as they relate to generalizability. The incorporation of radiation at the discretion of the treating physician added an additional confounding variable into the surgical toxicity and outcomes assessment, however, this mimics routine clinical practice and can be accounted for in future studies. Exploring potential biomarkers and molecular assays to tailor treatment approaches and maximize patient benefit from neoadjuvant NALIRIFOX is ongoing, specifically, circulating and tumor-specific biomarkers. Similarly, survival analyses are still ongoing with median and landmark 2-year OS planned to be reported at a future date. Collectively, these results provide benchmarks for future studies.

In conclusion, our study suggests that neoadjuvant NALIRIFOX is a viable and active therapy for R/BR PDAC, offering clinically meaningful response rates and very manageable toxicity. The clinically significant downstaging and a high R0 resection rate without excessive toxicity highlights its potential to improve surgical and survival outcomes in PDAC. Further follow-up to include survival outcomes and ongoing research to explore biomarkers of response will add more dimension to the data and will be subsequently reported. Additional studies that leverage these data and build on the use of NALIRIFOX along with treatment response biomarkers in the neoadjuvant treatment paradigm for PDAC are eagerly awaited, including as part of the next generation of TNT regimens.

## Supplementary Material

oyag250_Supplementary_Data

## Data Availability

The data supporting the findings of this study are available from the corresponding author upon reasonable request. Due to the inclusion of protected health information and institutional review board restrictions, the data are not publicly available.
