# Supplementary material for: Use of liposomal irinotecan with 5-FU and oxaliplatin (NALIRIFOX) in neoadjuvant pancreatic adenocarcinoma: NEO-Nal-IRI trial
Source: Oncologist. 2026 Jun 29;31(9):oyag250. doi: 10.1093/oncolo/oyag250 (PMC13451086; doi:10.1093/oncolo/oyag250)
Supplement: Supplementary file 1 [file oyag250_supplementary_data.zip › SupplementalFigureLegends.docx]

**Supplemental Figure S1. Quality of Life Assessment**

**Supp Fig S1.** Assessed using the Functional Assessment of Cancer Therapy - General (FACT-G), a validated patient reported outcome instrument. Mean and 95% confidence interval of QOL scores at each timepoint. EWB: Emotional Well-Being; FWB: Functional Well-Being; PWB: Physical Well-Being; SWB: Social/Family Well-Being; TOT: Total Score.

**Supplemental Figure S2. Postoperative major complication**

**Supp Fig S2.** Complications occurring within 30 days after surgery and contributing to the primary endpoint are defined as hospital re-admission, death, second surgery or interventional procedure, or major complications extending hospital stay. The overall complications were evaluated and sent by PI from each center. The confidence interval was calculated by the binomial exact test.

**Supplemental Figure S3. Radiographic Response/Clinical Benefit Rate**

**Supp Fig S3.** Left graph- radiographic response rate defined as proportion of patients achieving PR + CR. Right graph- Clinical benefit rate defined as proportion of patients achieving CR + PR + SD. In radiographic response rate, NE was not included for calculation. CR: Complete Response; PR: Partial Response; SD: Stable Disease; PD: Progressive Disease; NE: Not evaluated.
